# A Comparison of Characterization and Its Actions on Immunocompetent Cells of Polysaccharides from Sijunzi Decoction

**DOI:** 10.1155/2019/9860381

**Published:** 2019-11-29

**Authors:** Beibei Gao, Ying Peng, Chongsheng Peng, Yulong Zhang, Xiaobo Li

**Affiliations:** School of Pharmacy, Shanghai Jiao Tong University, Shanghai, China

## Abstract

Sijunzi decoction (SJZD) is a classic recipe in Traditional Chinese Medicine (TCM) with strong immune-enhancement activity. To further understand the characterization and immunomodulatory effect of polysaccharides from SJZD, the monosaccharide compositions of crude polysaccharide (SJZDP), polysaccharide fraction (S-3), and homogeneous polysaccharide (S-3-AG) from SJZD were compared by GC analysis, as well as their immunomodulatory effects on Peyer's patch cells, splenocytes, and macrophages which are related to intestinal immunity, specific immunity, and nonspecific immunity. The results showed that S-3-AG mainly contained Ara with a proportion of 38.9%, while Glc accounted for the largest proportion in S-3 (55.6%) and SJZDP (87.6%). The SJZDP, S-3, and S-3-AG all showed strong capability to stimulate Peyer's patch cells to proliferate and produce IgA and promoted the proliferation and IFN-*γ* production of splenocytes and increased the NO production and TNF-*α* production of macrophages. However, S-3 and S-3-AG were able to stimulate splenocytes to secret IL-4, SJZDP had no effect on IL-4 production of splenocytes in the tested concentrations. In addition, S-3 could stimulate the phagocytic activity of macrophages, and S-3-AG restrained the proliferation of macrophages at the concentration of 50–200 *µ*g/mL. These results suggested that SJZDP, S-3, and S-3-AG might have different immunomodulatory effects on intestinal immunity, specific immunity, and nonspecific immunity due to their different monosaccharide compositions. It will provide references for the material basis and mechanism of SJZD immunomodulation activity.

## 1. Introduction

Herbal products are becoming popular as alternative medicines worldwide. Traditional Chinese Medicine (TCM) has focused on this issue as its therapies have been validated and show potential clinical benefits. TCM prescription is usually a formula of several single herbs that are combined at a fixed ratio and boiled in water to form the decoction. Sijunzi decoction (SJZD), which is a classic recipe of TCM to strengthen the spleen and replenish Qi, has been widely applied to cure spleen deficiency [1]. Its ingredients include four Chinese herbs, the root of *Panax ginseng* C. A. Mey, the rhizome of *Atractylodes macrocephala* Koidz, the sclerotium of the fungus *Poria cocos* (Schw.) Wolf, and the root and rhizome of *Glycyrrhiza uralensis* Fisch in the ratio of 9 : 9 : 9 :6. Spleen deficiency is often accompanied by immune disorder [2], and modern pharmacological studies have shown that SJZD could strengthen the immune system [3, 4]. Chinese herbal compound prescriptions are often decocted with water, and polysaccharides are considered as the most abundant water-soluble ingredients in SJZD. Many studies have demonstrated that crude polysaccharides of SJZD (SJZDP) were the major effective component in SJZD [5, 6], which could restore immunomodulation function of immune damage models. For example, the function of immune organ/tissue (such as spleen and intestinal tissue), the ratio of immune cells (such as CD4^+^/CD8^+^), and cytokine production (such as IL-2 and IgA) were restored after oral administration of SJZDP in cyclophosphamide-induced immune injury mice [7], chemotherapy-treated tumor-bearing mice [8], and spleen-deficiency mice [5]. There are also reports of polysaccharides from SJZD ingredients such as crude drugs, Ginseng [9, 10], Rhizoma Atractylodis Macrocephalae [11], Poria [12], and Radix Glycyrrhizae [13] with immune-modulating activities, which supported the immunomodulation function of SJZDP. However, the systematic report about the extraction, isolation, purification, structure characteristics and immunomodulation activity of SJZDP and its fractions are limited.

Our previous study has indicated that S-3, the immunocompetent polysaccharide fraction screened from SJZDP could enhance the immune function of spleen-deficiency rats [14] by restoring the disturbance of gut microbiota and increasing the content of short-chain fatty acids. Furthermore, we isolated and purified an immune-modulating polysaccharide (S-3-1) from the S-3 fraction [14, 15] and found that the chemical composition of this polysaccharide and sugar residue connection were different from seven homogeneous polysaccharides from four crude drugs (Radix Ginseng, Rhizoma Atractylodis Macrocephalae, Poria, and Radix Glycyrrhizae) of SJZD using the same preparation method [16]. Recently, we isolated and purified a new water-soluble polysaccharide (S-3-AG) from the S-3 fraction; the information on the conformation of S-3-AG is required, and its structure-activity relationships were unclear.

A large number of studies have shown that the immunomodulating effect of polysaccharides in Chinese herbal medicine could be activated by stimulating effector cells such as intestinal lymphocytes in intestinal immunity [17–19], spleen lymphocytes [20], and macrophages in systemic immunity [21, 22]. SJZDP was found to enhance the specific immune function by acting on spleen lymphocytes [8, 23]. It is also the active component contributing to the function of intestinal immunoregulation, which can activate immunological response in peyer's patch [24, 25], mesenteric lymph nodes [26], intestinal epithelial cells [6], and intestinal intraepithelial lymphocytes [7]. And polysaccharides from four crude drugs of SJZD were demonstrated with macrophage immunomodulatory activities [16]. In order to further explore the immunomodulation activity of SJZDP and its fractions, homogeneous polysaccharide S-3-AG was purified from S-3. The structural characterizations of SJZDP, S-3, and S-3-AG were investigated, and their immunomodulatory effects on Peyer's patch (PP) cells, splenocytes, and macrophages were studied to assess their activity on intestinal immunity, specific immunity, and nonspecific immunity, respectively. This study provided references for the material basis and mechanism of SJZD immunomodulation activity.

## 2. Materials and Methods

### 2.1. Animals and Cell Lines

Male BALB/c mice aged 6–8 weeks were purchased from Beijing Vital River Laboratory Animal Technology Co., Ltd. (SPF certificate no. 11400700227651), bred, and housed under a standard laboratory condition with free access to food and water. All experimental protocols described in the study were approved by the Animal Ethical Committee of Shanghai Jiao Tong University.

The RAW 264.7 macrophage cell line was obtained from the Cell Bank of Shanghai Institutes for Biological Sciences, Chinese Academy of Sciences (Shanghai, China).

### 2.2. Herbs and Reagents

SJZD was prepared according the ratio of 3 : 3 : 3 : 2 by weight of four herbs, the root of *Panax ginseng* C. A. Mey, the rhizome of *Atractylodes macrocephala* Koidz, the sclerotium of the fungus, *Poria cocos* (Schw.) Wolf, and the root and rhizome of *Glycyrrhiza uralensis* Fisch. The herbs were bought from Shanghai Huayu Pharmaceutical Co., Ltd. (Shanghai, China) and authenticated by the corresponding author. Voucher specimens (RS001, BZ001, FL001, and GC001) were deposited at the herbarium of School of Pharmacy, Shanghai Jiao Tong University, Shanghai, China.

DEAE-Cellulose 52 and Sephadex G-100 were purchased from Solarbio Science & Technology Co., Ltd. Trifluoroacetic acid (TFA), dextrans, monosaccharide standards, dimethyl sulfoxide (DMSO), 3-(4,5-dimethyl-2-thiazolyl)-2,5-diphenyl-2-H-tetrazolium bromide (MTT), neutral red, lipopolysaccharide (LPS), and concanavalin A (ConA) were obtained from Sigma-Aldrich (Saint Louis, Missouri, USA). DMEM and RPMI-1640 cell culture medium were purchased from HyClone (South Logan, USA). Fetal bovine serum (FBS) and Hank's balanced salt solution (HBSS) were obtained from Gibco (Grand Island, USA). Assay kit for nitric oxide and ELISA kits (IgA, IL-4, IFN-*γ*, and TNF-*α*) were purchased from Nanjing Jiancheng Bioengineering Institute (Nanjing, China). All other chemicals used in this study were of analytical and cell culture grade.

### 2.3. Extraction and Purification of Polysaccharides from SJZD

SJZDP and S-3 were prepared based on reference [14]. In brief, dried herbs were boiled twice in distilled water and concentrated to 1 g/mL. The supernatant was centrifuged, and ethanol was then added to precipitate the crude polysaccharide. The precipitate was collected by centrifugation and deproteinated by the Sevage method. The crude polysaccharide precipitate was shaken vigorously with chloroform and n-butanol (4 : 1) for five times and washed with ethanol, acetone, and ether sequentially. Finally, the total polysaccharides (SJZDP) were dissolved in distilled water and lyophilized. SJZDP was dissolved and filtered through 0.45 mm filters, put into a DEAE-52 cellulose chromatography column and eluted with deionized water, 0.1 M NaCl, 0.2 M NaCl, and 0.3 M NaCl sequentially at the flow rate of 1 mL/min. The 0.3 M NaCl eluents were dialyzed in 3000 Da MWCO tubing in deionized water and concentrated to dryness, which named S-3. S-3 was then purified by gel filtration chromatography on a Sephadex G100 column (16 mm × 70 cm) and eluted with deionized water at a flow rate of 30 mL/h. The eluents were determined by high performance gel permeation chromatography (HPGPC) on a TSK-GEL G4000PW × l column (7.5 × 300 mm, Tosoh Co., Japan). The eluents whose retention time was 4.8 min with single and symmetrically shark peaks were combined, concentrated, and dried; the residue was collected and named S-3-AG. The homogeneity and molecular weight of fraction S-3-AG were evaluated and determined by high-performance gel permeation chromatography (HPGPC) on a TSK-GEL G4000PW × 1 column as described previously [16].

### 2.4. Monosaccharide Composition and Physicochemical Characterization Analyses

Monosaccharide composition analyses were determined by GC-MS as previously described [16]. In brief, polysaccharides (5 mg) were hydrolyzed separately with 2 M CF_3_COOH (4 mL) for 6 h at 100°C in a sealed glass tube and concentrated to dry residue by rotary evaporation. To remove CF_3_COOH, the residue was washed by methanol four times. Acetylation was carried out with 6 mg inositol, 10 mg hydroxylamine hydrochloride, and 1 mL pyridine by heating in a water bath for 30 min at 90°C. After incubation, the tubes were removed from the heat block, allowed to cool to room temperature, and then 1 mL of acetic anhydride was added and mixed thoroughly by vortexing. The tubes were sealed and incubated in a water bath shaker set at 90°C for 30 min again to produce alditol acetate derivatives, which were analyzed by an Agilent 7890A-5975C GC-MS (Agilent, USA) equipped with a total ionization detector (TIC) and a DB-5MS capillary column (30 m × 0.25 mm × 0.25 *μ*m, Agilent). The flow rate of carrier gas (He) was set at 1 mL/min. The temperature of the column oven was programmed as follows: (1) 100°C for 1 min; (2) increasing to 190°C at 20°C/min; and (3) increasing to 300°C at 10°C/min. Total carbohydrate content was determined by phenol-sulfuric acid colorimetric method using glucose as the standard [27]. The protein content was measured by the Lowry method [28] with BSA as the standard. The uronic acid content was measured by photometry with m-hydroxydiphenyl at 525 nm using d-galacturonic acid as the standard [29].

### 2.5. Structural Characterization of S-3-AG

The organic functional groups of S-3-AG were identified by the Fourier transform infrared (FT-IR) method. S-3-AG (1-2 mg) was dried in vacuum prior to grinding with spectroscopic grade KBr powder and then pressed into a pellet for FT-IR measurement by a Thermo Fisher Nicolet 6700 FT-IR spectrometer [30].

Periodate oxidation and Smith degradation were performed according to the method as previously described [30]. S-3-AG was oxidized with 15 mM NaIO_4_ and kept in the dark at 4°C. At 12 h intervals, 30 *μ*L aliquots were taken, diluted 250 times with distilled water, and read by spectrophotometry at 223 nm. The amount of NaIO_4_ consumed was calculated according to the NaIO_4_ standard curve. Production of formic acid was determined by titration. The remaining periodate product was treated with ethylene glycol and reduced with NaBH_4_. The dialysate was collected and concentrated to dryness. Then, methanol was added and evaporated to dryness for four times. The resulting product was hydrolyzed with triluoroacetic acid and aldononitrile acetate derivatives were prepared to GC-MS analysis.

Methylation analyses were performed as previously described [30]. S-3-AG was methylated with CH_3_I in dimethyl sulfoxide in the presence of dry sodium hydroxide. Distilled water was added to stop the reaction, and the methylated sample was then extracted with methyl dichloride. The methylene chloride extract was separated by centrifugation and evaporation. Methyl derivatives were prepared subsequently as follows: hydrolyzing the premethylated polysaccharide sample by treatment with 88% formic acid solution at 100°C for 3 h and 2 M TFA at 100°C for 6 h. The hydrolysate was reduced with 5% NaBH_4_ at room temperature for 4 h and acetylation with Ac_2_O at 100°C for 1 h. The disappearance of the OH band in the FT-IR spectrum was used to confirm complete methylation. The resulting methylated derivatives were detected by GC-MS.

Dried polysaccharide sample (20 mg) was dissolved in D_2_O, and ^13^C and ^1^H NMR spectra were recorded with a Bruker AMX-600 NMR spectrometer using sodium trimethylsilyl propionate (TSP) as the internal standard.

### 2.6. Measurements of Immunomodulatory Activity on Peyer's Patches (PPs)

PPs were collected from small intestinal tissue of BALB/c mice, and PP cells were extracted as previously described [31]. The PP cells were resuspended at a density of 1 × 10^7^ cells/mL in the RPMI-1640 medium containing 100 U/mL penicillin-streptomycin, 10% FBS, and 5 × 10^−5^ mol/L 2-mercaptoethanol. The suspension was seeded into 96-well plates (1 × 10^6^ cells/well) and treated with the 100 *µ*L RPMI-1640 medium or various concentrations of SJZDP, S-3, and S-3-AG samples (12.5, 50, and 200 *µ*g/mL), respectively, for 72 h. LPS (10 *µ*g/mL, a mitogen of B cells) and Con A (10 *µ*g/mL, a mitogen of T cells) were used as positive controls. After incubation, the proliferation of PP cells was determined by MTT assay [10]. The levels of immunoglobulin A (IgA) and interleukin-4 (IL-4) in cells culture supernatants were quantified using ELISA kits.

### 2.7. Measurements of Immunomodulatory Activity on Splenocytes

The preparation of mouse splenocyte suspension was performed as described previously [32]. The splenocytes were resuspended at a density of 1 × 10^7^ cells/mL in the DMEM medium, containing 100 U/mL penicillin-streptomycin and 10% FBS. The suspension was seeded into 96-well plates (1 × 10^6^ cells/well) and treated with 100 *µ*L DMEM or various concentrations of SJZDP, S-3, and S-3-AG samples (12.5, 50, and 200 *µ*g/mL), respectively, for 48 h. Con A (10 *µ*g/mL) was used as a positive control. After incubation, the proliferation of splenocytes was determined by MTT assay [10]. The levels of cytokines, interferon-*γ* (IFN-*γ*), and IL-4, in splenocyte culture supernatants were measured using ELISA kits.

### 2.8. Measurements of Immunomodulatory Activity on RAW 264.7 Cells

RAW 264.7 cells were seeded in a 96-well plate (5 × 10^4^ cells/well) and treated with 100 *µ*L DMEM medium and various concentrations of SJZDP, S-3 and S-3-AG samples (12.5, 50, and 200 *µ*g/mL), respectively, for 48 h. LPS (5 *µ*g/mL) was used as a positive control. After incubation, the proliferation of macrophages was determined by MTT assay [10]. The phagocytic ability of macrophages was determined by neutral red uptake [10]. Nitrite accumulation was measured using Griess reagent [10]. An ELISA kit was used to determine the level of tumor necrosis factor-*α* (TNF-*α*) production.

### 2.9. Statistical Analyses

All statistical analyses were performed using the Statistical Package for Social Sciences (SPSS) version 19.0 (SPSS Inc., USA). The differences between the control and the treatments in these experiments were evaluated by one way analysis of variance (ANOVA) and were considered significant at *p* < 0.05. All data are presented as mean ± standard deviation (SD).

## 3. Results

### 3.1. Structural Characterizations of SJZDP, S-3, and S-3-AG

**Figure 1:**
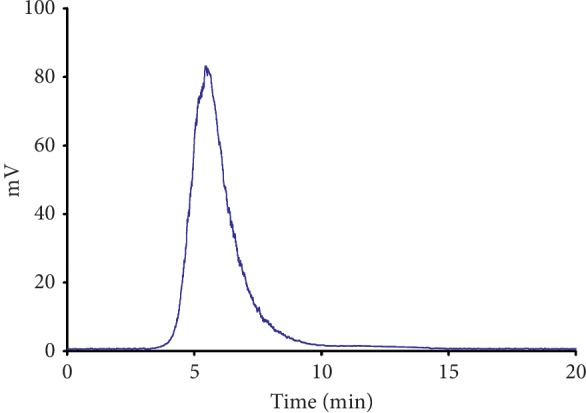
HPGPC chromatogram of S-3-AG obtained with a TSK-GEL G4000PW × 1 column (7.5 × 300 mm, Tosoh), eluted with ultrapure water at 1.0 mL/min, and detected by ELSD.

The monosaccharide compositions of SJZDP and S-3-AG were determined by GC analysis and compared with that of S-3 [14]. The results showed that all the three polysaccharides were composed of Rha, Ara, Xyl, Man, Glc, and Gal with different molar ratios. S-3-AG mainly contained Ara with a proportion of 38.9%, while Glc accounted for the largest proportion in S-3 (55.6%) and SJZDP (87.6%), respectively. Higher proportions of Ara and Gal were obtained after purification from SJZDP to S-3-AG. As shown in Table 1, the total sugar contents of SJZDP, S-3, and S-3-AG determined by the phenol-sulfuric acid colorimetric method were 84.15%, 67.5%, and 97.8%, respectively. The percentages of uronic acid of SJZDP, S-3, and S-3-AG were 13.97%, 10%, and 18.5%, respectively, and the total protein contents determined by the Lowry method were decreased after purification. The HPGPC profile suggested that S-3-AG was homogeneous (Figure 1), as it showed a symmetrical peak in HPGPC. According to the retention time, the average molecular weight of S-3-AG was estimated as 44.3 × 10^4^ Da.

Furthermore, the structural characterization of this homogeneous polysaccharide S-3-AG was performed. The FT-IR spectra of S-3-AG revealed polysaccharide-specific absorption bands ([Supplementary-material supplementary-material-1]). The strong broad absorption peak at 3420 cm^−1^ was due to the hydroxyl stretching vibration of the polysaccharide, and the peak at 2920 cm^−1^ was due to the C-H stretching vibration band. The peak at 1624 cm^−1^ was attributed to the bending vibration of C=O, and the signal at 1450 cm^−1^ band was attributed to the variable angling vibration of C-H. The characteristic absorption band at 1150 cm^−1^, 1070 cm^−1^, and 1040 cm^−1^ suggested the presence of pyranoside configurations in S-3-AG.

The locations of glycosidic linkages in the S-3-AG are preliminarily determined by periodate consumption and production of formic acid in periodate oxidation. The results from periodate oxidation showed that per 10 mg polysaccharide sample (0.059 mmol sugar residue), 0.3729 mmol of periodate was consumed, and 0.1695 mmol formic acid was produced. The production of formic acid indicated that some of the residue may exist in the pyranose 1,2-linked, 1,2,6-linked, 1,4-linked, and 1,4,6-linked forms, since the amount of periodate consumed was more than twice the amount of formic acid produced. The periodate-oxidized products of S-3-AG were further reduced, hydrolyzed, and analyzed by GC-MS. The results showed that the degradation process completely removed glucose residues, indicating that the glycoside residues may exist in the 1,2-linked, 1,2,6-linked, or 1,4-linked, 1,4,6-linked forms, which could be oxidized. A large proportion of glycerol were probably from 1,2-linkages, 1,6-linkage, 1,2,6-linkage glucose or 1,5-linkage pentose. And, erythritol probably indicated 1,4-linkages and 1,4,6-linkages pyranose.

From [Supplementary-material supplementary-material-1], based on the results of the methylation analysis on S-3-AG, the following conclusions can be drawn: (1) the Ara residues were present as 1,5-linked and 1,3-linked Araf residues; (2) the Gal residues were present as 1,6-linked Galp residues; (3) the Galacturonic acid residues were present as 1,5-linked GalA residues; (4) the Glc residues were present as terminal, 1,4-linked Glcp residues; (5) the Man residues were present as 1,2,3-linked, 1,3,6-linked Manp residues; (6) the Rha residues were present as 1,2-linked Rhap residues.

600-MHz ^1^H-NMR and 150-MHz ^13^C-NMR spectra of S-3-AG are shown in [Supplementary-material supplementary-material-1]. The ^1^H and ^13^C-NMR spectra of polysaccharide S-3-AG were crowded in narrow regions ranging from 3.0 to 5.5 ppm (^1^H NMR) and 60 to 110 ppm (^13^C NMR) which were typical of polysaccharides. The ^1^H NMR signals at 1.2533 ppm were attributed to the methyl group of Rha, and the signals at 5.4069 ppm indicated the existence of furanose in S-3-AG. The ^13^C NMR signals at 69.90 ppm were assigned as the 1,6-linked galactopyranose residues configuration; the signals at 70.68 ppm were assigned to C-6 substituted glucopyranose residues; the signals at 80.82 ppm were assigned as the 1,4-linked glucopyranose residues; the signals at 99.48 ppm, 99.56 ppm, and 103.31 ppm were assigned to *α*-Manp residues; the signals at 107.41 ppm were assigned to C-1 in *α*-1,5-arabinofuranose residues; the signals at 109.26 ppm, 81.21 ppm, 83.85 ppm, 83.80 ppm, and 61.04 ppm were assigned as C-1 to C-5 in *α*-1,3-arabinofuranose residues; the signals at 174.86 ppm were assigned to the carbonyl carbon of uronic acid; the signals at 16.79 ppm were assigned to the methyl group of Rha. These results suggested that the backbone of S-3-AG might be composed of 1,5-linked Araf residues, with branches of the 1,3-linked Araf, 1,6-linked Galp, 1,5-linked GalA, 1,4-linked Glcp, 1,2,3-linked, and 1,3,6-linked Manp, 1,2-linked Rhap, and terminal Glcp.

### 3.2. Immunomodulation Activities of SJZDP, S-3, and S-3-AG on PP Cells

**Figure 2:**
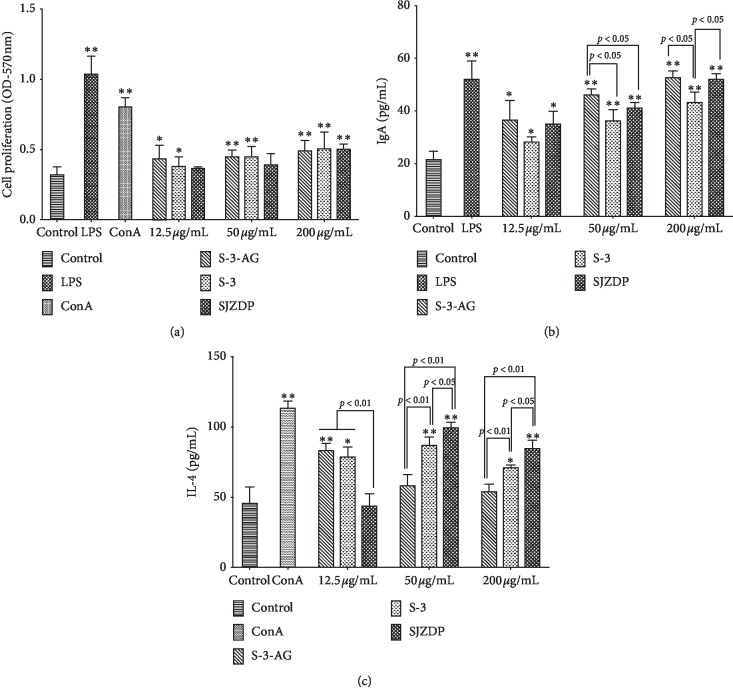
Immunomodulatory activity of SJZDP, S-3, and S-3-AG on Peyer's patch (PP) cells. Adherent PP cells were incubated with various concentrations of polysaccharides (12.5, 50, and 200 *µ*g/mL) for 72 h. The DMEM medium was used as the blank control. LPS (10 *µ*g/mL) and ConA (10 *µ*g/mL) were used as positive controls. (a) Effects of S-3-AG, S-3, and SJZDP on PP cell proliferation. (b) Effects of S-3-AG, S-3, and SJZDP on IgA production of PP cells. (c) Effects of S-3-AG, S-3, and SJZDP on IL-4 production of PP cells. Each value was expressed as mean ± SD (*n* = 3). ^*∗*^indicates *p* < 0.05; ^*∗∗*^indicates *p* < 0.01 compared with the blank control.

The effects of SJZDP, S-3, and S-3-AG on proliferation, IgA, and IL-4 production of PP cells were investigated to assess their intestinal immunomodulation. As shown in Figure 2(a), at the concentration range of 12.5–200 *µ*g/mL, S-3-AG and S-3 significantly promoted the proliferation of PP cells (*p* < 0.05 for 12.5 *µ*g/mL, *p* < 0.01 for 50 *µ*g/mL and 200 *µ*g/mL), whereas SJZDP only promoted the proliferation of PP cells significantly at a concentration of 200 *µ*g/mL (*p* < 0.01). No significant difference was found among the effects of SJZDP, S-3, and S-3-AG on PP cell proliferation at the same concentration, ranging from 12.5 *µ*g/mL to 200 *µ*g/mL. Moreover, SJZDP, S-3, and S-3-AG could significantly stimulate PP cells to produce IgA in a dose-dependent manner as shown in Figure 2(b). Interestingly, S-3-AG has a significantly stronger effect on IgA production of PP cells than S-3 at 50 *µ*g/mL and 200 *µ*g/mL (*p* < 0.05), S-3-AG stimulated IgA production more significantly than SJZDP at 50 *µ*g/mL (*p* < 0.05). As shown in Figure 2(c), SJZDP, S-3, and S-3-AG could induce the IL-4 production of PP cells. Although IL-4 is one of the key cytokines that induce IgA production, the increases of IL-4 induced by three polysaccharides were not correlated with the IgA production. All concentrations of S-3 showed a significant increase in IL-4 production, whereas S-3-AG significantly induced the IL-4 production (*p* < 0.01) only at the concentration of 12.5 *µ*g/mL. And, SJZDP remarkably induced the IL-4 production at the concentration of 50–200 *µ*g/mL (*p* < 0.01). At a lower level concentration (12.5 *µ*g/mL), S-3-AG and S-3 had a stronger effect on IL-4 production of PP cells than SJZDP (*p* < 0.01). But at the concentration range from 50 *µ*g/mL to 200 *µ*g/mL, it was found that SJZDP showed a more significant promotion effect on IL-4 production than S-3 (*p* < 0.05) and S-3-AG (*p* < 0.01). The results indicated that SJZDP, S-3, and S-3-AG could promote the proliferation of PP cells and induced the IgA and IL-4 production of PP cells, showing intestinal immunomodulatory activity. However, their activity on the IgA and IL-4 production of PP cells was different and dose dependent.

### 3.3. Immunomodulation Activities of SJZDP, S-3, and S-3-AG on Splenocytes

**Figure 3:**
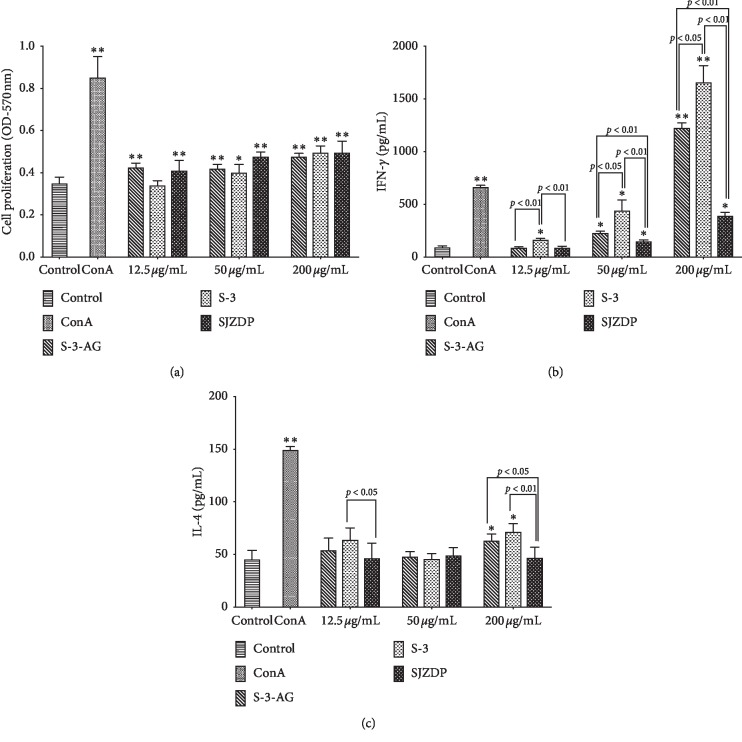
Immunomodulatory activity of SJZDP, S-3, and S-3-AG on splenocytes. Adherent splenocytes were incubated with various concentrations of polysaccharides (12.5, 50, and 200 *µ*g/mL) for 48 h. The DMEM medium and ConA (10 *µ*g/mL) were used as the blank and positive control, respectively. (a) Effects of S-3-AG, S-3, and SJZDP on splenocyte proliferation. (b) Effects of S-3-AG, S-3, and SJZDP on IFN-*γ* production of splenocytes. (c) Effects of S-3-AG, S-3, and SJZDP on IL-4 production of splenocytes. Each value was expressed as mean ± SD (*n* = 3). *∗*indicates *p* < 0.05; ^*∗∗*^indicates *p* < 0.01 compared with the blank control.

The activities of SJZDP, S-3, and S-3-AG on specific immunomodulation were assessed by their effects on proliferation, IFN-*γ* production, and IL-4 production of splenocytes. As shown in Figure 3(a), SJZDP and S-3-AG at the concentration of 12.5–200 *µ*g/mL promoted the proliferation of splenocytes significantly (*p* < 0.01), and S-3 significantly promotes the proliferation at concentrations over 50 *µ*g/mL (*p* < 0.05). SJZDP-, S-3-, and S-3-AG-induced IFN-*γ* production of splenocytes were in a dose-dependent manner as shown in Figure 3(b). We observed that S-3 at all tested concentrations, S-3-AG and SJZDP at the concentration over 50 *µ*g/mL could significantly improve the IFN-*γ* production of splenocytes (*p* < 0.05). In addition, 200 *µ*g/mL of S-3-AG and S-3 showed marked stimulation effects on IFN-*γ* production, which were 1.84 and 2.49 times of ConA (a mitogen of T cells), as well as 13.05 and 17.62 times of the control group, respectively. The comparison results of activity on IFN-*γ* production between S-3-AG, S-3, and SJZDP were also shown in Figure 3(b). S-3 showed the most obvious activity on promoting IFN-*γ* production at all tested concentrations. And, S-3-AG had a more significant promotion effect than SJZDP at the concentration starting from 50 *µ*g/mL. As described in Figure 3(c), no significant promotion effect on IL-4 secretion was found except S-3-AG and S-3 at the concentration of 200 *µ*g/mL (*p* < 0.05) compared with the control group. At the concentration of 200 *µ*g/mL, S-3-AG and S-3 showed significantly higher activity than SJZDP (*p* < 0.05), and they were 1.39 and 1.58 times of the control group. Although there was no significant difference found compared with the control group at 12.5 *µ*g/mL, the IL-4 promotion effect of S-3 was more significant than SJZDP (*p* < 0.05). Taken together, the results indicated that SJZDP, S-3, and S-3-AG could promote the proliferation and IFN-*γ* production of splenocytes, in which S-3 showed the strongest effect. S-3 and S-3-AG were capable of stimulating splenocytes to secret IL-4 slightly, whereas SJZDP had no effect on IL-4 production at all tested concentrations. The effects of SJZDP, S-3, and S-3-AG on IFN-*γ* (Th1-type cytokine) seem to be stronger than that on IL-4 (Th2-type cytokine).

### 3.4. Immunomodulation Activities of SJZDP, S-3, and S-3-AG on Macrophages

**Figure 4:**
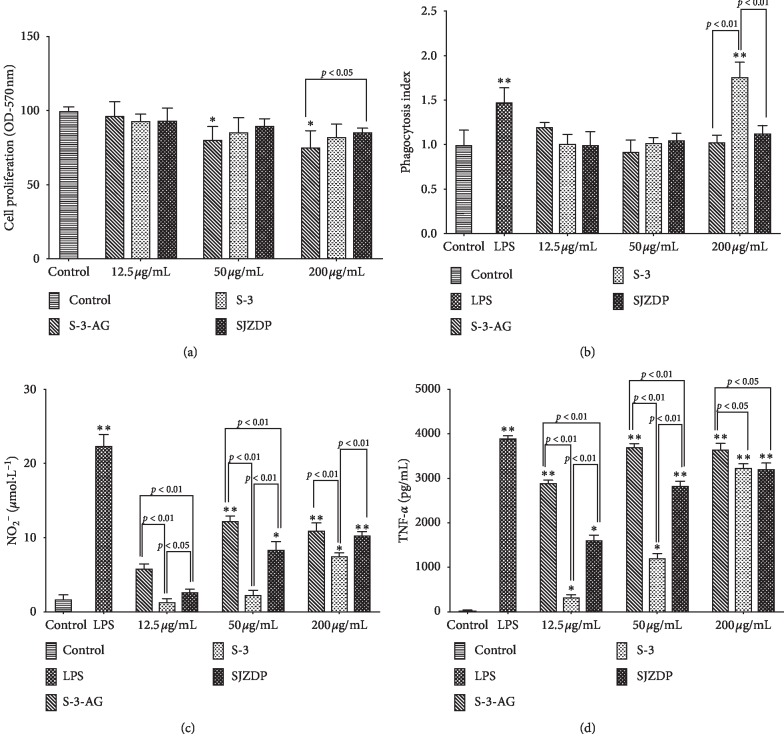
Immunomodulatory activity of SJZDP, S-3, and S-3-AG on RAW 264.7 cells. Adherent RAW 264.7 macrophages were incubated with various concentrations of polysaccharides (12.5, 50, and 200 *µ*g/mL) for 48 h. The DMEM medium and LPS (5 *µ*g/mL) were used as the blank and positive control. (a) Effects of S-3-AG, S-3, and SJZDP on RAW 264.7 cell viability. (b) Effects of S-3-AG, S-3, and SJZDP on phagocytic activity. (c) Effects of S-3-AG, S-3, and SJZDP on NO production. (d) Effects of S-3-AG, S-3, and SJZDP on TNF-*α* production. Each value was expressed as mean ± SD (*n* = 3). ^*∗*^indicates *p* < 0.05; ^*∗∗*^indicates *p* < 0.01 compared with the blank control.

The activities of SJZDP, S-3, and S-3-AG on nonspecific immunomodulation were assessed by their effects on proliferation, phagocytic activity, NO, and TNF-*α* production of macrophages. As shown in Figure 4(a), except that the proliferation of macrophages was significantly suppressed by higher concentration (>50 *µ*g/mL) of S-3-AG (*p* < 0.05), no significant effect was found compared with the control group. In addition, S-3 was found to, at the concentration of 200 *µ*g/mL, significantly promote the phagocytic activity of macrophages (*p* < 0.01) (Figure 4(b)). No significant positive or negative effect on macrophage phagocytic activity was found in other groups. The effects of the three polysaccharides on NO production are presented in Figure 4(c). The results showed that S-3-AG and SJZDP could significantly promote the NO production with concentrations higher than 50 *µ*g/mL (*p* < 0.05), and S-3 could only significantly promote the NO production (*p* < 0.05) with a concentration higher than 200 *µ*g/mL. S-3-AG and SJZDP showed a significantly higher promotion effect on NO production than S-3 at the same level (from 12.5 *µ*g/mL to 200 *µ*g/mL, *p* < 0.05). As shown in Figure 4(d), SJZDP, S-3, and S-3-AG at all tested concentrations could induce the TNF-*α* production of macrophages. The stimulation of TNF-*α* production induced by S-3-AG at the concentration of 50–200 *µ*g/mL was at a similar level to the LPS-treated (positive control) group. For the comparison between S-3-AG, S-3, and SJZDP of the same concentration, S-3-AG exhibited significantly higher activity on TNF-*α* production of macrophages than S-3 and SJZDP at all tested concentrations. At the concentration of 12.5–50 *µ*g/mL, a significant difference between S-3 and SJZDP in the TNF-*α* promotion activity on macrophages (SJZDP showed a higher activity) is demonstrated in Figure 4(d), which was eliminated at a higher concentration (200 *µ*g/mL). These results showed that S-3-AG at the concentration of 50–200 *µ*g/mL restrained the proliferation of macrophages and only high-level (200 *µ*g/mL) S-3 could significantly stimulate the phagocytic activity of macrophages. In the case of NO production and TNF-*α* production, S-3-AG had the highest promoting effect at all tested concentrations.

## 4. Discussion

The immunologic activity of polysaccharides from herbal medicines can be initiated by activating the effector cells such as intestinal lymphocytes of gut immunity [17–19], splenic lymphocytes, [20] and macrophages [16, 22] of systemic immunity. SJZD is a classic recipe of TCM to strengthen the spleen and replenish Qi [1]. Spleen deficiency is closely related to disorders of the immune system [2]. In our study, we found that SJZDP, S-3, and S-3-AG all had the intestinal immunomodulatory activity, S-3 performed the best on specific immune system, and both S-3 and S-3-AG had the nonspecific immunomodulatory activity through immunocompetent cell tests *in vitro*. These findings imply that S-3-AG and S-3 may be used as candidates for developing immunomodulating agents in food and pharmaceutical industries. Previous studies showed that the disturbance of gut microbiota induced by spleen deficiency could be restored after administration of SJZDP [5] and S-3 [14]. As intestinal microbiota plays an important role in gut immunity, further *in vivo* study is needed to investigate whether the different immunomodulatory activities of SJZDP, S-3, and S-3-AG are caused by their regulation of different intestinal bacteria and their different monosaccharide compositions.

The small intestinal PP was one of the important gut-associated lymphoreticular tissues. IgA plays a critical role as an immunological barrier in the intestine, which is the predominant intestinal immunoglobulin and acts as the first line of defense for the intestinal mucosa. This antibody inhibits microbial adherence and prevents the absorption of antigens into mucosal surfaces [33]. Moreover, essential cytokines, such as IL-4, TGF-*β*, GM-CSF, IL-6, and IL-10, enhance intestinal IgA production by promoting the differentiation of IgA-expressing B cells into plasma cells [34–36]. We found that SJZDP, S-3, and S-3-AG significantly increased the IgA production of PP cells in a dose-dependent manner, which indicates the high intestinal immune protection activity of these three polysaccharides. However, their activity on the IgA and IL-4 production of PP cells were different. It may be explained by different structural characteristics. S-3-AG has the stronger effect on IgA production than SJZDP and S-3, indicating the purification process of polysaccharides enhanced their activity on IgA production of PP cells. These may be related to higher proportions of Ara and Gal obtained after purification from SJZDP to S-3-AG. Moreover, not all the effects of these three polysaccharides on IL-4 production were correlated with the IgA production. These results suggested that SJZDP and its subfractions may activate different cytokines to promote the differentiation of IgA-expressing B cells. Further studies are needed to explore if SJZDP and its fractions, especially S-3-AG, induce the IgA production by stimulating cytokines besides IL-4, such as TGF-*β* and GM-CSF. In the intestinal immune activity study of crude polysaccharide isolated from Korean persimmon vinegar (KPV-0) [19], the production of IgA was elicited by TGF-*β* and IL-6 with KPV-0 treatment, whereas GM-CSF production was unaffected by KPV-0 treatment in PP cells *in vitro*.

As the biggest peripheral immune organ, the spleen plays a significant role in specific immunity, contributing to both cell-mediated and humoral immunity [37]. SJZDP, S-3, and S-3-AG could promote the proliferation of splenocytes which is one of the most important steps in the activation pathway of host immunity [38]. The ability of promoting splenic cell proliferation has been widely used as a method to screen for new immunostimulators [39]. Therefore, SJZDP, S-3, and S-3-AG all have the potentiality to be a new immunostimulator. Furthermore, it was found that these three polysaccharides were capable of inducing the IFN-*γ* production, which was similar to the activity of extracellular polysaccharide fraction A-g obtained after gel filtration from *G. lucidum* polysaccharide [40]. It was notable that S-3 showed the strongest effect on IFN-*γ* production of splenocytes, which differs from the results of PP cells. This may be due to the fact that there are other active polysaccharides in S-3, except S-3-AG. In addition, Th1 lymphocytes produce IL-2 and IFN-*γ* that orchestrate cell-mediated immunity [41] against intracellular pathogens [37] and tumors [42]. IL-4 is secreted by Th2 cells and plays an important role in humoral immunity [43]. The effects of SJZDP, S-3, and S-3-AG on IFN-*γ* were stronger than those on IL-4, indicating these three polysaccharides may mainly take effects on Th1-mediated immunity in the specific immunity, which was similar to *G. lucidum* polysaccharide [40, 44] and sulfated *Lentinus edodes α*-(1 → 3)-D-glucan [45].

Moreover, SJZDP, S-3, and S-3-AG significantly improved the TNF-*α* production and NO production of macrophages, in which S-3-AG had the highest promoting effect. NO production has been linked with many biological functions, including vasodilatation, neurotransmission, immune response, and platelet aggregation [46]. TNF-*α* plays an important role in the cellular immune process by aiding the elimination of abnormal cells [47], which is related to the antitumor activity [42]. Polysaccharides such as *Cynanchum wilfordii* polysaccharide [48] and red gensing polysaccharide [10] had been proved to have macrophage stimulation function. In our previous study, it was found that three homogeneous polysaccharides, RS-3-1, BZ-3-1, and FL-3-1, which are obtained from three herbal medicines of SJZD (Ginseng Radix, Atractylodes, Macrocephalae Rhizoma, and Poria) could activate the phagocytic activity of macrophages at the concentration over 500 *µ*g/mL [16]. In our study, we found that phagocytosis was not enhanced by S-3-AG from 12.5 to 200 *µ*g/mL. This may be explained by the reduced phagocytic activity inhibited by S-3-AG (as the proliferation of macrophages was significantly suppressed by 50 and 200 *µ*g/mL concentration of S-3-AG). Whether the suppression effect of S-3-AG on the proliferation of macrophages was similar to *Portulaca oleracea* L. polysaccharide [49], whose mechanism may be associated with the sub-G1 phase cell cycle arrest, remained to be verified. In addition, although the production of macrophage NO and TNF-*α* (which are key factors for an increase in phagocytosis) was highly improved by SJZDP than S-3, we failed to demonstrate the phagocytosis enhanced by SJZDP at tested concentrations. An increase in phagocytosis was only observed at a concentration of 200 *µ*g/mL S-3. It was known that macrophage NO was mainly synthesized by inducible nitric oxide synthase (iNOS) [50], which was an important effector molecule to induce macrophage phagocytosis. However, excessive NO can promote macrophage to release inflammatory factors and aggravate inflammatory reaction [51]. Therefore, moderate inhibition of NO secretion can also improve macrophage phagocytosis [52]. This may be the reason that phagocytosis was enhanced by S-3, whereas not by SJZDP and S-3-AG. Moreover, S-3-AG exhibited significantly higher activity on TNF-*α* production of macrophages than S-3 and SJZDP at all tested concentrations, which may be related to the suppressed proliferation of macrophages.

## 5. Conclusion

In the present study, SJZDP, S-3, and S-3-AG isolated from SJZD were demonstrated to have immune-enhancing effects *in vitro*. SJZDP, S-3, and S-3-AG had the intestinal immunomodulatory activity. S-3 performed best on specific immune system, and both S-3 and S-3-AG had the nonspecific immunomodulatory activity. These findings suggested that SJZDP, S-3, and S-3-AG had different immunomodulation activities, which may be owing to different compositions. The results would provide important references for the material basis and immunomodulation mechanism of SJZD.

## Figures and Tables

**Table 1:** Physicochemical property and monosaccharide compositions of SJZDP, S-3, and S-3-AG.

Fraction	Total sugar (%)	Protein (%)	Uronic acid (%)	Mw (Da)	Rha	Ara	Xyl	Man	Glc	Gal
S-3-AG	97.8	—	18.5	44.3 × 10^4^	3.9	38.9	0.8	1.0	22.1	33.4
S-3 [8]	67.5	11.2	10	NA	7.3	10.1	2.6	5.2	55.6	19.1
SJZDP	84.15	14.5	13.97	NA	0.8	5.8	0.3	1.1	87.6	4.4

—, not detected; NA, not analyzed.

## Data Availability

The data used to support the findings of this study are included within the article.

## Abbreviations

Ara: Arabinose
BSA: Bovine serum albumin
ConA: Concanavalin A
DMSO: Dimethylsulfoxide
ELSD: Evaporative light-scattering detector
FBS: Fetal bovine serum
Gal: Galactose
GC: Gas chromatography
GC-MS: Gas chromatography-mass spectrometer
Glc: Glucose
GM-CSF: Granulocyte-macrophage colony-stimulating factor
HBSS: Hank's balanced salt solution
HPGPC: High-performance gel permeation chromatography
IFN-*γ*: Interferon-*γ*
IgA: Immunoglobulin A
IL-2: Interleukin-2
IL-4: Interleukin-4
IL-6: Interleukin-6
IL-10: Interleukin-10
KPV: Korean persimmon vinegar
LPS: Lipopolysaccharide
Man: Mannose
MTT: 3-(4,5-dimethyl-2-thiazolyl)-2,5-diphenyl-2-H-tetrazolium bromide
Mw: Weight average molecular weight
NO: Nitric oxide
PP: Peyer's patch
Rha: Rhamnose
RPMI-1640: Roswell Park Memorial Institute-1640
SJZD: Sijunzi decoction
SJZDP: Crude polysaccharide of SJZD
TCM: Traditional chinese medicine
TFA: Trifluoroacetic acid
TGF-*β*: Transforming growth factor-*β*
Th1: Type 1 helper T cells
Th2: Type 2 helper T cells
TNF-*α*: Tumor necrosis factor-*α*
Xyl: Xylose.

## Supplementary Materials

Supplementary Materials Figure S1: infrared spectrum of S-3-AG. Figure S2: total ion flowchart of methylation of S-3-AG: (1) 2,3,4,6-Me4-Glc; (2) 4,6- Me2-Man; (3) 2,3,4- Me3-Gal; (4)2-O-Me-6-deoxy-GalA; (5) 2,3,6- Me3-Glc; (6) 3,4-Me2-Rha; (7) 2,5-Me2-Ara; (8) 2,3-Me2-Ara; (9) 2,3,4- Me3-Gal; (10) 2,4- Me2-Man. Figure S3: 1 H NMR spectrum of S-3-AG in D2O (600 MHz) (A) and 13 C NMR spectrum of S-3-AG in D2O (125 MHz) (B).
