# Supplementary material for: A Comparison of Characterization and Its Actions on Immunocompetent Cells of Polysaccharides from Sijunzi Decoction
Source: Evid Based Complement Alternat Med. 2019 Nov 29;2019:9860381. doi: 10.1155/2019/9860381 (PMC6935442; doi:10.1155/2019/9860381)
Supplement: Supplementary Materials — Figure S1: infrared spectrum of S-3-AG. Figure S2: total ion flowchart of methylation of S-3-AG: (1) 2,3,4,6-Me4-Glc; (2) 4,6- Me2-Man; (3) 2,3,4- Me3-Gal; (4)2-O-Me-6-deoxy-GalA; (5) 2,3,6- Me3-Glc; (6) 3,4-Me2-Rha; (7) 2,5-Me2-Ara; (8) 2,3-Me2-Ara; (9) 2,3,4- Me3-Gal; (10) 2,4- Me2-Man. Figure S3: 1 H NMR spectrum of S-3-AG in D2O (600 MHz) (A) and 13 C NMR spectrum of S-3-AG in D2O (125 MHz) (B). [file 9860381.f1.doc]

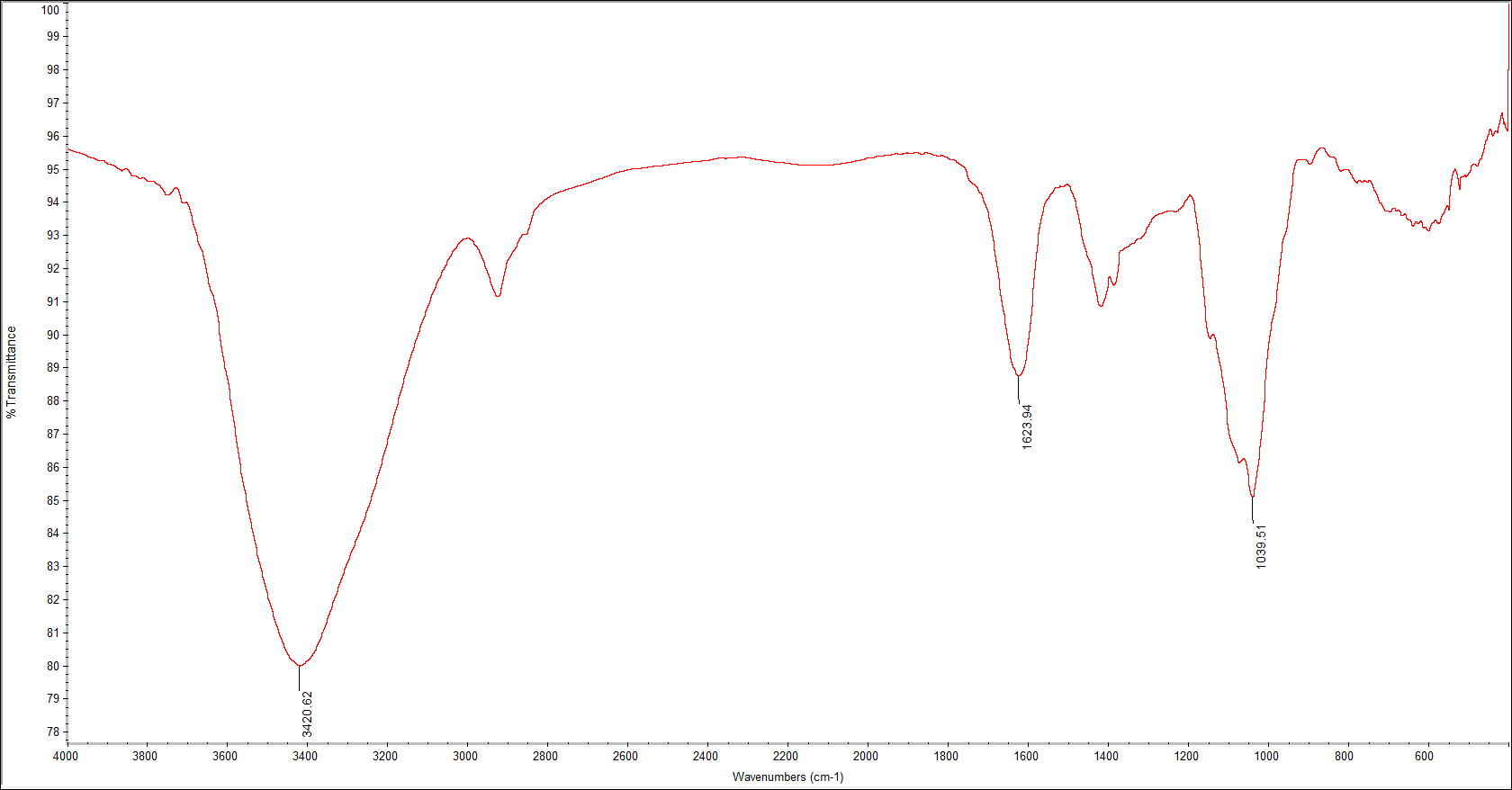


Figure S1 Infrared spectrum of S-3-AG

Figure S2 Total ion flow chart of methylation

(1)2,3,4,6-Me4-Glc (2)4,6- Me2-Man (3)2,3,4- Me3-Gal (4) 2-O-Me-6-deoxy-GalA (5) 2,3,6- Me3-Glc (6)3,4-Me2-Rha (7) 2,5-Me2-Ara (8) 2,3-Me2-Ara (9)2,3,4- Me3-Gal (10) 2,4- Me2-Man


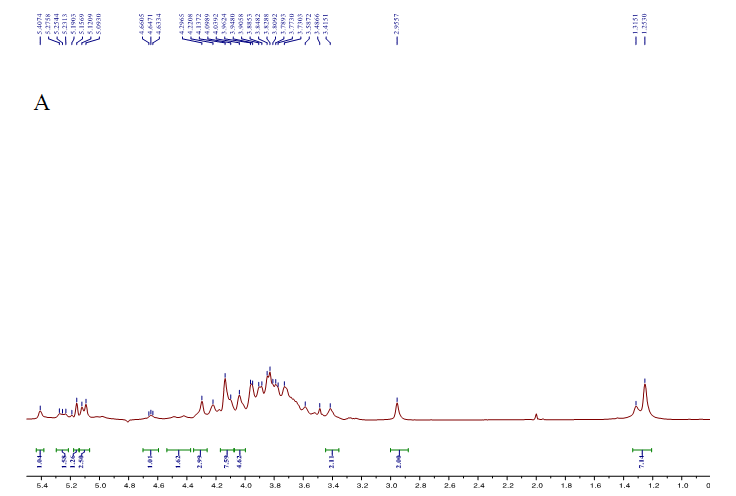


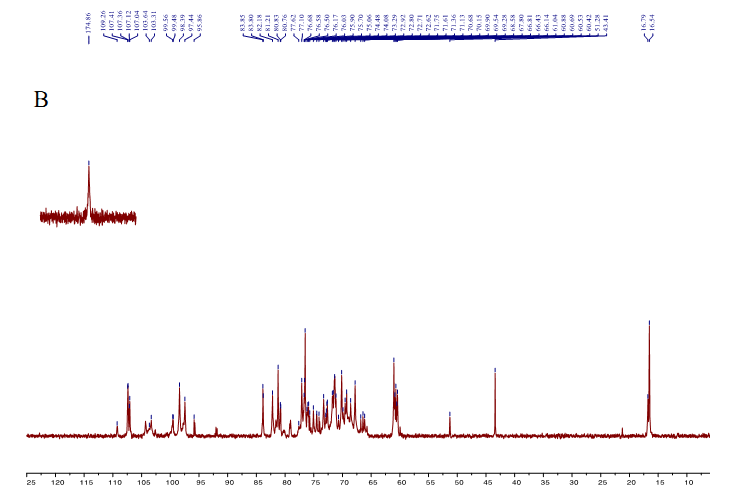


Figure S3 ^1^ H NMR NMR spectrum of S-3-AG in D_2_O (600 MHz) (A), ^13^ C NMR spectrum of S-3-AG in D_2_O (125 MHz) (B) .
